# Artificial Diets Based on Selective Amino Acid Restriction versus Capecitabine in Mice with Metastatic Colon Cancer

**DOI:** 10.3390/nu14163378

**Published:** 2022-08-17

**Authors:** Julio José Jiménez-Alonso, Emilio Guillén-Mancina, José Manuel Calderón-Montaño, Víctor Jiménez-González, Patricia Díaz-Ortega, Estefanía Burgos-Morón, Miguel López-Lázaro

**Affiliations:** Department of Pharmacology, Faculty of Pharmacy, University of Seville, 41012 Sevilla, Spain

**Keywords:** anticancer activity, colorectal cancer, metastasis, selective amino acid restriction therapy, cancer metabolism, amino acids, cysteine, glutamine, leucine

## Abstract

New therapies are needed to improve the low survival rates of patients with metastatic colon cancer. Evidence suggests that amino acid (AA) restriction can be used to target the altered metabolism of cancer cells. In this work, we evaluated the therapeutic potential of selective AA restriction in colon cancer. After observing anticancer activity in vitro, we prepared several artificial diets and evaluated their anticancer activity in two challenging animal models of metastatic colon cancer. These models were established by injecting CT26.WT murine colon cancer cells in the peritoneum (peritoneal dissemination) or in the tail vein (pulmonary metastases) of immunocompetent BALB/cAnNRj mice. Capecitabine, which is a first-line treatment for patients with metastatic colon cancer, was also evaluated in these models. Mice fed diet TC1 (a diet lacking 10 AAs) and diet TC5 (a diet with 6% casein, 5% glutamine, and 2.5% leucine) lived longer than untreated mice in both models; several mice survived the treatment. Diet TC5 was better than several cycles of capecitabine in both cancer models. Cysteine supplementation blocked the activity of diets TC1 and TC5, but cysteine restriction was not sufficient for activity. Our results indicated that artificial diets based on selective AA restriction have therapeutic potential for colon cancer.

## 1. Introduction

Colon cancer is one of the most common cancers worldwide [1]. In recent years, improvements in diagnosis, surgery, and pharmacotherapy have increased the survival rates of patients with this type of cancer. However, the prognosis is highly dependent on the initial stage of diagnosis; survival rates are high when the disease is diagnosed in the early stages, but decrease dramatically when metastasis occurs. The 5-year survival rate for stage IV colon cancer patients is 14.7% [2].

Approximately 20.9% of patients with colon cancer have metastatic disease at the time of diagnosis [3]. Liver, lung, and peritoneal metastases are the most common places for metastases in patients with colon cancer [4]. When cancer cells have already spread to distant organs, surgery is no longer curative. At this stage of the disease, pharmacotherapy becomes the main treatment for these patients. Currently, several antineoplastic drugs are used for colorectal cancer, including cytotoxic drugs such as 5-fluorouracil, capecitabine, oxaliplatin, and irinotecan. New targeted therapies with angiogenesis inhibitors and EGFR inhibitors are also used. Immunotherapy is also active against certain subtypes of colon cancer with defective DNA repair mechanisms (MSI-H and dMMR) [5]. The new anticancer drugs have improved overall survival in patients but rarely cure metastatic disease.

The main weakness of most anticancer drugs is their narrow selectivity toward cancer cells; the drug doses required to kill all the cancer cells in a patient would also kill their normal cells. Therefore, patients receive tolerable doses rather than effective doses, which are not enough to cure the disease in most cases. Increasing selectivity toward cancer cells is key to improving the survival rate in patients with metastasis. Finding major and exploitable differences between cancer cells and normal cells can lead to more selective anticancer therapies [6].

Cancer cells and normal cells are metabolically different [7]. The metabolic alterations of cancer cells were first observed by Otto Warburg almost a century ago and are recognized today as one of the hallmarks of cancer [8]. There are several relevant metabolic alterations in cancer cells, including increased aerobic glycolysis (Warburg effect) [9], dependence on the external intake of non-essential AAs (NEAAs) [10,11], altered lipid metabolism [12], increased nucleotide biosynthesis [13], and increased oxidative stress [14]. These metabolic adaptations support the survival and proliferation of cancer cells.

In recent years, efforts have been made to target the altered AA metabolism in cancer cells [10], including colon cancer cells. Dietary and pharmacological restriction of the sulfur-containing AAs methionine (Met) and cysteine (Cys) have shown anticancer effects in colon cancer [15,16,17,18,19]. Cys restriction can reduce intracellular glutathione levels and trigger ferroptotic cell death [20]. Dietary and pharmacological restriction of serine (Ser) and glycine (Gly), which are needed in the one-carbon cycle, have also shown anticancer activity in intestinal cancers [21,22,23,24,25,26]. Dietary modulation of arginine (Arg) has also shown activity in models of colon cancer [27,28,29]. A dietary restriction of AAs is becoming a feasible novel strategy for targeting colon cancer cells.

In this work, we evaluated the therapeutic potential of selective AA restriction in colon cancer. After observing that an artificial medium lacking 10 AAs induced selective anticancer activity in vitro, we prepared several artificial diets with major changes in AA levels and ratios and evaluated their anticancer activity in two challenging animal models of metastatic colon cancer. Two artificial diets improved mice survival, and one of the diets was better than the first-line anticancer drug capecitabine in both animal models.

## 2. Materials and Methods

### 2.1. Cell Lines and Cell Culture Conditions

The CT26.WT cell line (murine colon cancer) was obtained from American Type Culture Collection (ATCC). The HaCaT cell line (human skin keratinocytes) was purchased from Cell Lines Service (CLS) [30]. The HT29 cell line (human colon cancer) was generously provided by Dr. Helleday (Karolinska Institute, Sweden). The HT29 and HaCaT cell lines were cultured in Dulbecco’s modified Eagle’s medium (DMEM) high glucose medium. The CT26.WT cell line was grown in RPMI 1640. All media were supplemented with 100 U/mL penicillin, 100 µg/mL streptomycin, and 10% fetal bovine serum. All cells were kept at 37 °C in a humidified atmosphere containing 5% CO_2_. Cell culture reagents were purchased from Biowest or Thermo Fisher Scientific.

### 2.2. Chemicals and Drugs

MTT, SDS, and 5-fluorouracil (5-FU, F6627) were purchased from Sigma. AAs were obtained from different sources, including Applichem and Acros Organics. Casein protein (27607; bovine), choline bitartrate (450225000), and tert-butylhydroquinone (TBHQ, 150822500) were obtained from Acros Organic. Sucrose was obtained from local markets, salmon oil from Petspurest, Mineral Mix (AIN-93M-MX) and Vitamin Mix (AIN Vitamin Mixture 76) from MP Biomedicals, corn starch and cellulose from Farmusal (local pharmacy), and India Ink (Superblack India Ink, Speedball, 33 × 089A) from Amazon. Capecitabine (500 mg/tablet, Normon) and sterile physiological serum were obtained from a local pharmacy.

### 2.3. In Vitro Experiments

To test the anticancer activity of selective AA restriction in vitro, artificial cell media were prepared in our laboratory using powdered DMEM without AAs (D9800-13; US Biological). Media were completed with glucose and specific AAs. Each AA was previously dissolved in PBS and sterilized via microfiltration. All media were completed with 10% FBS and 1% penicillin/streptomycin. Table 1 shows the compositions of the media used in this work.

Human colon cancer cells (HT29), human non-malignant cells (HaCaT), and mouse colon cancer cells (CT26.WT) were seeded in 96-well plates in complete DMEM. After 24 h, the medium was removed and replaced with an AA-manipulated medium (M1), complete DMEM (M0), or complete DMEM with several concentrations of the anticancer drug 5-FU. The culture media were changed every three days to avoid exhaustion of nutrients and excessive acidification of the medium. Cells were visualized under a microscope and photographed (20× magnification) using a Huawei P9 lite Leica camera adapted to an inverted microscope. Three days of treatment followed by 4 days of recovery in a standard medium (7 days in total) were sufficient to observe a potent cytotoxic effect in cells exposed to 5-FU. Seven days of treatment were necessary to observe a marked cytotoxic effect in cells treated with our AA-manipulated medium. The cell viability was estimated at these times with the MTT assay.

The MTT assay is a redox-based colorimetric technique based on the capacity of metabolically viable cells to reduce the MTT reagent (3-(4,5-dimethylthioazol-2-yl)-2,5-diphenyltetrazolium bromide) into an insoluble purple formazan product. The number of viable cells is directly proportional to the amount of final product formed [31]. To perform the MTT assay, the medium was removed and 125 µL MTT (1 mg/mL in standard medium) was added to each well. Four hours later, 80 µL of 20% SDS in 0.02 M HCl was added to the wells. The plates were incubated overnight at 37 °C before measuring optical densities at 540 nm using an absorbance spectrophotometer microplate reader. Results were expressed as a percentage of cell viability relative to untreated cells grown in their standard media. Cell viability was expressed as a percentage in relation to controls. Data were averaged from at least two independent experiments.

### 2.4. Animals

All mice were purchased from Janvier Labs^®^ (Le Genest-Saint-Isle, France). Female BALB/cAnNRj mice (12 weeks or older) were used in all experiments. The animals were kept in our animal laboratory facilities for at least two weeks before starting the experiments to allow for acclimatization. They were kept under standard conditions (24 °C, 70–75% humidity, 12 h light/12 h dark cycles, with ad libitum access to food and water). During this time, mice continued to be fed with their standard diet (ssniff diet R/M-Z E/R/S; V1724-000, ssniff Spezialdiäten). All in vivo experiments were approved by the Animal Ethics Committee of the University of Seville (CEEA-US2018-6/2 and CEEA-US2019-20) and Junta de Andalucía (15/05/2018/090 and 13/11/2020/131). They were conducted under the recommendations of the European Union regarding animal experimentation (Directive of the European Parliament and of the Counsel 2010/63/EU).

### 2.5. In Vivo Colon Cancer Models

The peritoneal dissemination model was established by injecting 1 × 10^5^ CT26.WT murine colon cancer cells into the peritoneal cavity of BALB/cAnNRj mice. The lung metastasis colon cancer model was established by injecting 1 × 10^5^ CT26.WT murine colon cancer cells into the tail vein of BALB/cAnNRj mice. Inoculation of CT26.WT cells generates metastatic tumors in immunocompetent BALB/c mice [32].

Due to the aggressiveness of these models [32], treatments started 4 days after the cancer cell inoculation. One day before the beginning of the treatments, the mice were randomly distributed into several groups. In the control group (untreated animals), mice continued to be fed the standard diet (ssniff diet). In the positive control group, mice were treated with the maximum tolerated doses of the oral anticancer drug capecitabine by following a schedule of 7 days on and 7 days off (7/7) to maximize the anticancer activity of this drug [33]. To prepare the capecitabine treatment, we powdered the standard diet (ssniff diet) and added capecitabine at a dose of 2500 mg/kg diet; then, we added water to make a soft dough, which was pelleted and left air-dried until use. A 25 g mouse typically consumed an average of 4.5 g of diet per day, resulting in a dose of capecitabine of approximately 450 mg/kg/day. The mice received 2–3 cycles of capecitabine depending on their state of health, which was determined by the toxicity of the drug and the progression of the tumors. In the groups of mice treated with artificial diets based on selective AA restriction, the treatments simply consisted of replacing their normal diet with one of the artificial diets.

All treatments were scheduled to last 6 weeks (from day 4 after cancer cell injection to day 46). The animals were monitored daily and body weights were determined periodically (at least three times a week). The mice were sacrificed via cervical dislocation when signs of disease progression were apparent; these signs (for example, excessive gains or losses in body weight, reduced mobility and curiosity, respiratory distress, and/or visible or palpable tumors larger than 15–20 mm) indicated that survival for an additional 48 h was unlikely. A postmortem examination was performed to confirm the cause of death, observe the extent of the disease, and weigh and observed selected organs (Supplementary Figures S1 and S2). Unless otherwise stated or shown, autopsies confirmed the presence of tumors and similar tumor loads in all euthanized mice. Lungs were dyed with India ink to better observe tumors in the lungs. First, the trachea and lungs were excised from the chest cavity. The trachea was then cannulated with a 20 G needle, and the lungs were insufflated with India ink (1:5 in PBS). Finally, the lungs were immersed in Fekete’s solution (100 mL of 70% ethanol, 10 mL of 37% formaldehyde, and 5 mL of 100% glacial acetic acid). With this technique, tumors show a white appearance and normal lung parenchyma appears black.

To follow the recommendations of the Animal Ethics Committee and reduce the number of animals to a minimum, treatments were initially screened using 2–4 mice. Additional independent experiments with a higher number of animals were carried out to confirm the anticancer activity of the active treatments. Mice receiving the same treatment were merged in the survival curves to facilitate the comparison between groups. All experiments included a control group (untreated mice) and a positive control group (capecitabine 450 mg/kg/day).

### 2.6. Diet Preparation and Composition

The diets were prepared by mixing all the solid ingredients until they formed a well-blended dry powder. After adding the oil to the mixture, water was added gradually to make a soft dough. The dough was air dried for about 2 h, manually pelleted (approximately 5 g/pellet), air-dried for an additional 24 h, and stored until use. When Cys was added to diets, it was in the form of cystine (a dimer of Cys). Table 2 shows the composition of the artificial diets used in our work.

Casein (Acros Organics; 27607; bovine casein) provided AAs in diets TC5–TC8. The typical amounts (g) of AAs in 100 g and 6 g (shown in brackets) of the casein used in the experiments was as follows: Gln + Glu—21.7 (1.302), Leu—9 (0.54), Met—2.9 (0.174), Phe—4.8 (0.288), His—2.6 (0.156), Lys—7.5 (0.45), Thr—4.1 (0.246), Iso—4.3 (0.258), Val—5.3 (0.318), Trp—1.2 (0.072), Cys/CySS—0.7 (0.042), Arg—3.4 (0.204), Gly—1.7 (0.102), Ser—5.7 (0.342), Tyr—5.2 (0.312), Ala—2.9 (0.174), Asp + Asn—6.9 (0.414), and Pro—10.1 (0.606).

Mineral Mix (AIN-93M-MX, MP Biomedicals) constituted 3.5% of the dry diet. A 100 g portion of this diet contained 1.25% calcium carbonate, 0.875% monopotassium phosphate, 0.098% potassium citrate, 0.259% sodium chloride, 0.163% potassium sulfate, 0.085% magnesium oxide, 0.021% ferric citrate, 0.0058% zinc carbonate, 0.0022% manganese carbonate, 0.0011% copper carbonate, 0.000035% potassium iodate, 0.000035% sodium selenate, 0.000028% ammonium paramolybdate-tetrahydrate, 0.0051% sodium metasilicate-nonahydrate, 0.00095% chromium potassium sulfate-dodecahydrate, 0.0000595% lithium chloride, 0.000284% boric acid, 0.00022% sodium fluoride, 0.00011% nickel carbonate hydroxide, 0.000021% ammonium meta-vanadate, and 0.73% sucrose.

Vitamin Mix (AIN Vitamin Mixture 76, MP Biomedicals) made up 1% of the dry diet. A 100 g portion of dry diet contained (mg) thiamine hydrochloride (0.6), riboflavin (0.6), pyridoxine hydrochloride (0.7), nicotinic acid (3), D-calcium pantothenate (1.6), folic acid (0.2), D-biotin (0.02), cyanocobalamin (0.001), retinyl palmitate premix (250,000 IU/g) (1.6), DL-a-tocopherol acetate (250 IU/g) (20), cholecalciferol (400,000 IU/g) (0.25), menaquinone (0.005), and sucrose (972.9).

### 2.7. Statistical Analysis

Data are expressed as mean value ± standard error of the mean (SEM). GraphPad Prism version 7.0 software was used. Statistical analysis for the Kaplan–Meier survival curves was calculated using the Gehan–Breslow–Wilcoxon (GBW) test. The *t*-test (paired, two-tailed) was also used. A *p*-value < 0.05 is considered statistically significant and is indicated with an asterisk (*), <0.01 with two asterisks (**), and <0.001 with three asterisks (***).

## 3. Results

### 3.1. Selective Amino Acid Restriction Induced Anticancer Activity in Colon Cancer Cells In Vitro

We used a human colon cancer cell line (HT29), a murine colon cancer cell line (CT26.WT), and a highly proliferative human non-malignant cell line (HaCaT) to evaluate the anticancer potential of selective AA restriction in vitro. We followed a patient-oriented approach [6,31]; since cancer patients need more selective treatments, we evaluated whether the selectivity of our anticancer strategy was higher than the selectivity of a treatment of choice in metastatic colon cancer. Because capecitabine (an oral drug used as a first-line treatment) is a prodrug of 5-FU, we used 5-FU in the in vitro experiments.

**Figure 1:**
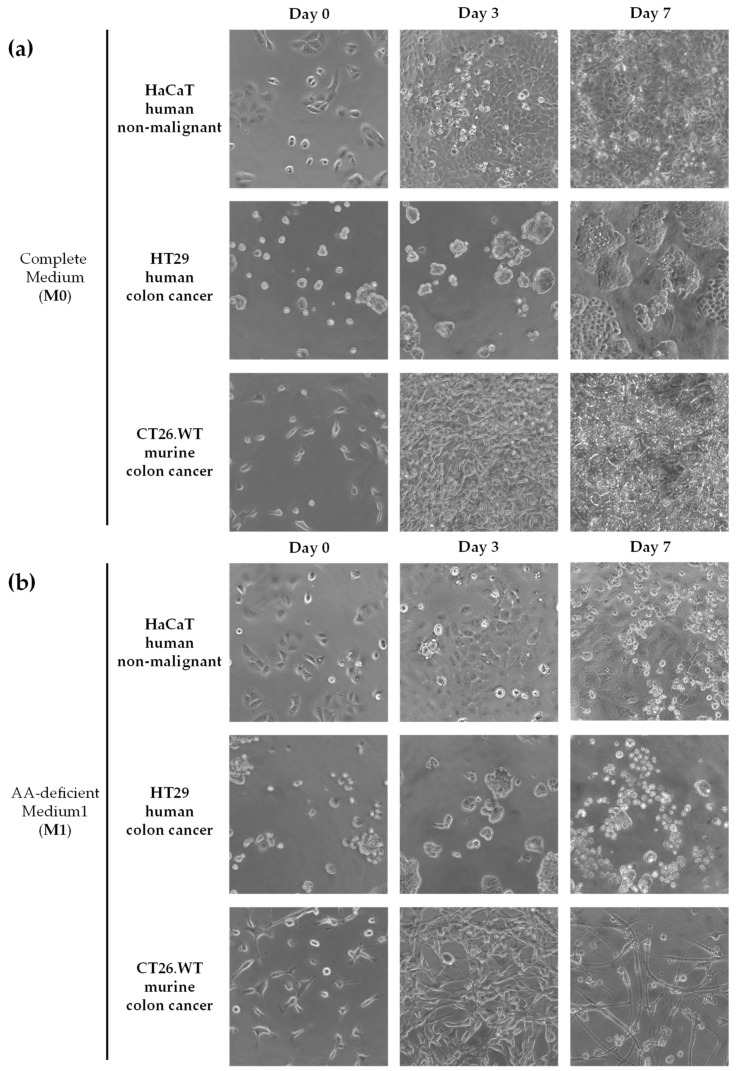
Anticancer activity of selective AA restriction. Representative photographs at 20× magnification are shown. The detailed composition of M0 (**a**) and M1 (**b**) are shown in Table 1.

**Figure 2:**
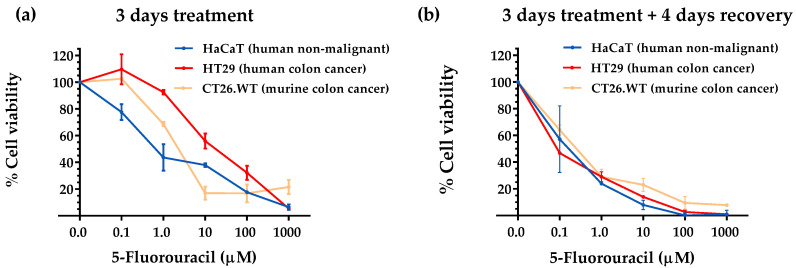
Cell viability of HaCaT, HT29, and CT26.WT cells treated with 5-FU. (**a**) Cell viability after 3 days of 5-FU treatment. (**b**) Cell viability after 3 days of 5-FU treatment followed by 4 days of recovery in DMEM. Cell viability was estimated with an MTT assay. All data are expressed as percentages relative to controls.

After carrying out the experimental protocol described in the Materials and Methods section, we observed that our artificial medium lacking 10 AAs induced cytotoxicity and selectivity against the human colon cancer cell line after 7 days of exposure. 5-FU was cytotoxic but lacked selectivity; the highly proliferative non-malignant cell line was equally affected by 5-FU treatment. Briefly, when comparing the images of the cell lines grown in a complete medium (M0, Figure 1a) with those grown in a medium lacking 10 AAs (M1, Figure 1b), we observed that medium M1 inhibited cell proliferation after 7 days of treatment. The antiproliferative effect of medium M1 was higher in the human colon cancer cell line (HT-29) than in the highly proliferative human non-malignant cell line (HaCaT). Evidence of cell death could also be observed in the HT-29 cells, but not in the HaCaT cells (Figure 1b). The MTT assay revealed that the viability percentages of the cells exposed to medium M1 on day 7 (relative to untreated cells grown in standard media) were 5.06 ± 2.44% in the HT-29 colon cancer cells and 30.37 ± 17.16% in the HaCaT non-malignant cells. Figure 2 shows that 5-FU was cytotoxic against the three cell lines; however, no selectivity was observed toward human cancer cells at any of the concentrations tested (0.1, 1, 10, 100, and 1000 µM).

### 3.2. An Artificial Diet Lacking 10 Amino Acids Induced Anticancer Activity in Mice with Colon Cancer

After observing that medium M1 inhibited colon cancer cell proliferation in vitro, we prepared an artificial diet lacking the same 10 AAs (diet TC1) to evaluate its anticancer activity in mice with colon cancer. As described in the Materials and Methods section, we used two challenging in vivo models, which were established by injecting CT26.WT murine colon cancer cells in the peritoneum (peritoneal dissemination) or in the tail vein (pulmonary metastases) of immunocompetent BALB/cAnNRj mice. In both models, the inoculation of 10^5^ CT26.WT cancer cells led to the development of multiple tumors in the animals (Supplementary Figures S3 and S4). All untreated animals died several weeks after the inoculation of the cancer cells. Capecitabine (450 mg/kg/day), which is a first-line treatment for patients with metastatic colon cancer, was also evaluated in these models.

**Figure 3:**
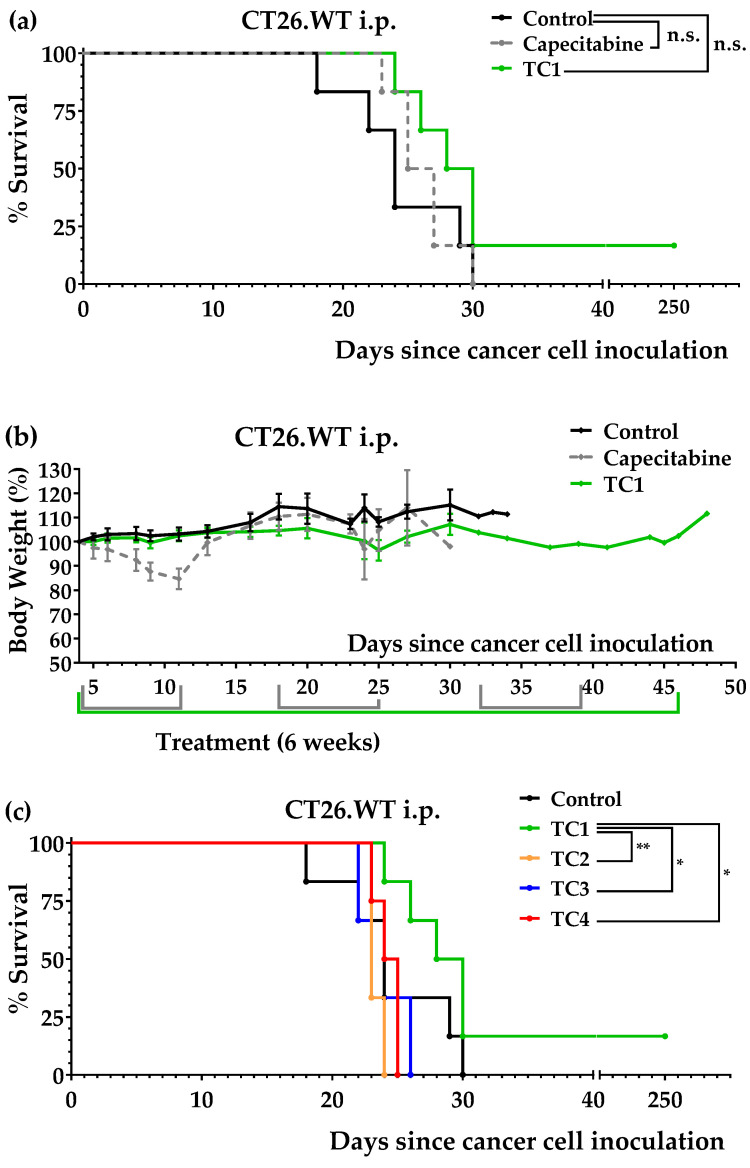
Anticancer activity of diets TC1, TC2, TC3, TC4, and capecitabine in mice with colon cancer (CT26.WT murine colon cancer cells inoculated in the peritoneum of immunocompetent BALB/c mice; peritoneal dissemination model). (**a**) Survival of the untreated mice (control), the mice treated with diet TC1 (normal diet was replaced by the TC1 diet for 6 weeks), and the mice treated with oral capecitabine (450 mg/kg/day, 7/7 schedule, 3 cycles). (**b**) Mice body weights (mean percentage ± SEM) relative to the body weights at the beginning of the treatments (day 4). (**c**) Comparison of survival of the mice fed with diets TC1, TC2, TC3, and TC4 over 6 weeks. The *p*-values were calculated with the GBW test; * indicates *p* < 0.05, and ** indicates *p* < 0.01. n.s. means no significance.

We initially used the peritoneal dissemination model to evaluate the anticancer activity of diet TC1. Figure 3a shows that the mice fed diet TC1 lived longer (mean survival ± SEM = 64.7 ± 37.1 days; *n* = 6) than the untreated mice (mean survival ± SEM = 24.5 ± 1.8 days; *n* = 6). Importantly, this diet was more effective than several cycles of capecitabine (mean survival ± SEM = 26.2 ± 1.0 days; *n* = 6). A mouse fed diet TC1 survived the treatment and was sacrificed on day 250; no signs of disease or tumors were found during the autopsy (Supplementary Figure S5). The TC1 diet was well tolerated by the mice, while capecitabine induced a significant weight loss that reverted at the end of each treatment cycle (Figure 3b).

Diets TC2, TC3, and TC4 were also tested in this colon cancer model. As shown in Table 2, diet TC2 was diet TC1 plus 0.2% Cys, diet TC3 was diet TC1 plus 0.2% Cys and a reduction in Leu levels, and diet TC4 was diet TC1 plus 0.2% Cys and a small reduction in Met levels. All three diets were completely inactive in the mice with colon cancer; the mice fed diet TC2 diet lived 23.3 ± 0.3 days, the mice fed diet TC3 lived 23.7 ± 1.2 days, and the mice fed diet TC4 lived 24.3 ± 0.5 days (Figure 3c). These results showed that the anticancer activity of diet TC1 was blocked by supplementation with a small percentage of Cys.

**Figure 4:**
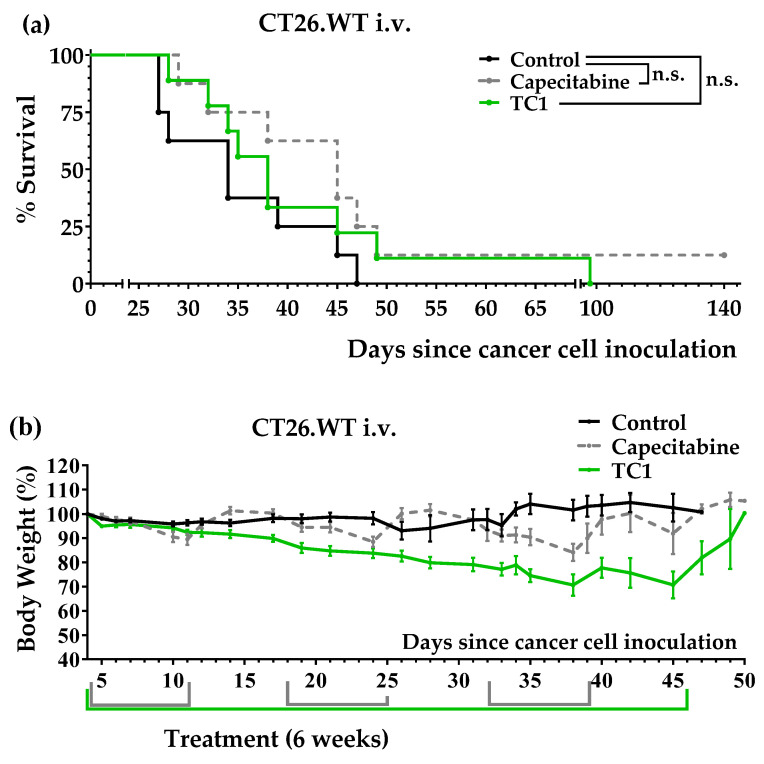
Anticancer activity of diet TC1 and capecitabine in mice with colon cancer (lung metastasis model; CT26.WT murine colon cancer cells inoculated in the tail vein of immunocompetent BALB/c mice). (**a**) Survival of the mice with colon cancer left untreated (control), treated with diet TC1 (6 weeks), or treated with capecitabine (450 mg/kg/day, 7/7 schedule, 3 cycles). (**b**) Body weights (mean percentage ± SEM) relative to the body weights at the beginning of treatments (day 4). The *p*-values were calculated with the GBW test. n.s. means no significance.

Next, we used the colon cancer model of lung metastasis to confirm the anticancer activity of diet TC1. In this model, several days after the inoculation of 10^5^ murine colon cancer cells into the tail vein of the mice, the cells formed numerous tumors in the lungs [32]. Treatments started four days after the tail vein injection. The untreated mice lived 35.1 ± 2.8 days (*n* = 8), the mice fed with diet TC1 lived 44.1 ± 7.1 days (*n* = 9), and the mice treated with capecitabine lived 53.1 ± 12.7 (*n* = 8) (Figure 4a). One of the mice treated with capecitabine survived the treatment; it was sacrificed on day 140 and no tumors were found during the autopsy (Supplementary Figure S6). The TC1 diet was well tolerated; however, the body weights decreased slowly but continually during treatment. Capecitabine induced weight losses that reverted at the end of each treatment cycle (Figure 4b). These results showed that the TC1 diet had anticancer activity in this colon cancer model; however, the standard treatment of capecitabine was better. In addition, the progressive weight loss observed in mice treated with diet TC1 in this model may limit the clinical translatability of this anticancer strategy.

### 3.3. An Artificial Diet with 6% Casein, 5% Glutamine, and 2.5% Leucine Induced Marked Anticancer Activity in Mice with Colon Cancer

Although diet TC1 (a diet lacking 10 AAs) showed anticancer activity in both colon cancer models (Figure 3 and Figure 4), two aspects may limit its clinical translatability. As shown in Figure 4b, mice progressively lost weight during the treatment in the tail vein model. In addition, the AA-formulated diets have poor organoleptic properties [34,35], which may lead to low compliance rates in clinical trials [34]. To overcome these limitations, we sought to optimize our artificial diets by incorporating small amounts of protein.

Because the addition of Cys blocked the anticancer activity of diet TC1 (Figure 3c), we selected the protein casein, which is known to contain very low levels of Cys. We used 6% casein, which provided approximately 0.042% Cys. The amount of Met provided by 6% casein is 0.17%, which is similar to the amount of this AA in diet TC1. Controlling the Met levels is important because Met can produce Cys through the transsulfuration pathway [36]. We prepared four different diets containing 6% casein: TC5–TC8 (Table 2). In diet TC6, casein was the only source of AAs. Diet TC5 contained 6% casein, 5% Gln, and 2.5% Leu; diet TC7 contained 6% casein, 5% Gln, and 5% Leu; and diet TC8 contained 6% casein, 5% Gln, 2.5% Leu, and 0.2% Cys.

**Figure 5:**
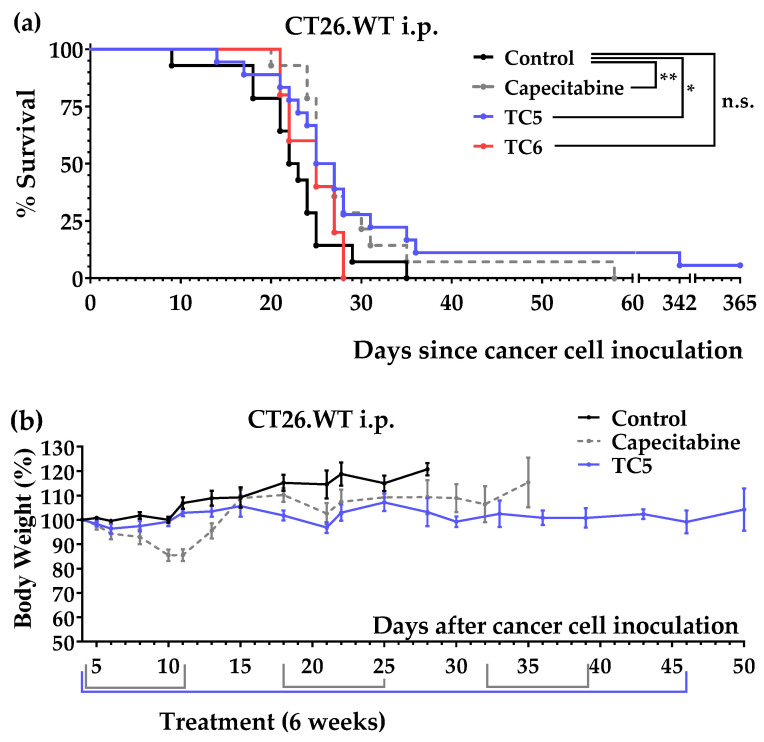
Anticancer effect of diet TC5 and capecitabine in mice with colon cancer (peritoneal dissemination model; CT26.WT murine colon cancer cells inoculated in the peritoneum of immunocompetent BALB/c mice). (**a**) Survival of the mice left untreated (control), treated with diet TC5 (normal diet was replaced by diet TC5 for 6 weeks), with diet TC6, or with oral capecitabine (450 mg/kg/day, 7/7 schedule, 3 cycles). (**b**) Body weights (mean percentage ± SEM) relative to the body weights at the start of the treatments (day 4). The *p*-values were calculated with the GBW test; * indicates *p* < 0.05, and ** indicates *p* < 0.01. n.s. means no significance.

We initially tested diet TC5 and diet TC6 in the intraperitoneal colon cancer model and observed that diet TC5 significantly increased mice survival (Figure 5a). The untreated mice lived 22.6 ± 1.6 days (*n* = 14), the mice fed diet TC5 lived 61.9 ± 25.1 days (*n* = 18), the mice fed diet TC6 lived 24.6 ± 1.4 days (*n* = 5), and the mice treated with several cycles of capecitabine lived 28.9 ± 2.4 days (*n* = 14). Two mice (from two independent experiments) treated with the TC5 diet survived a very long time. One of them was sacrificed on day 342, and the autopsy revealed a small (2 mm) metastatic tumor in the lungs. The other mouse was sacrificed on day 365; no sign of disease or tumors was observed (Supplementary Figure S7). Importantly, unlike capecitabine, diet TC5 was very well tolerated and the mice did not lose weight (Figure 5b). The inactivity of diet TC6 (whose only source of AAs was 6% casein) indicated that the presence of 5% Gln and 2.5% Leu in diet TC5 was important for anticancer activity.

**Figure 6:**
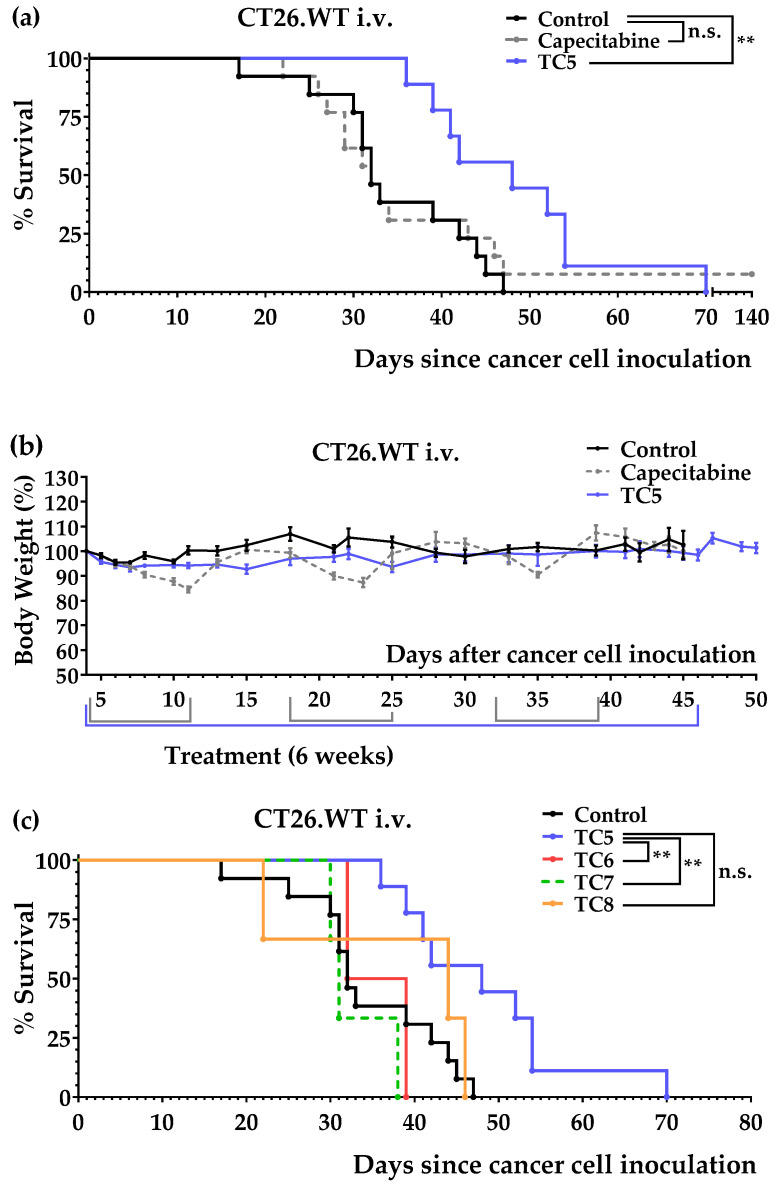
Anticancer activity of diets TC5, TC6, TC7, TC8, and capecitabine in mice with colon cancer (lung metastasis model; CT26.WT murine colon cancer cells inoculated in the tail vein of immunocompetent BALB/c mice). (**a**) Survival of the mice with colon cancer left untreated (control), treated with diet TC5 (6 weeks), or treated with capecitabine (450 mg/kg/day, 7/7 schedule, 3 cycles). (**b**) Body weights (mean percentage ± SEM) relative to the body weights of the mice at the beginning of treatment (day 4). (**c**) Comparison of survival of the mice fed for 6 weeks with diets TC5, TC6, TC7, and TC8. The *p*-values were calculated with the GBW test; ** indicates *p* < 0.01. n.s. means no significance.

We next used the tail vein colon cancer model to confirm the in vivo anticancer activity of diet TC5. We also tested diets TC6, TC7, and TC8 to further assess the effects of Cys, Gln, and Leu on the activity of diet TC5. The results, which are represented in Figure 6a, showed that diet TC5 significantly increased mice survival and that the activity was better than that observed in the mice treated with several cycles of capecitabine. The untreated mice lived 34.5 ± 2.4 days (*n* = 13), the mice fed diet TC5 lived 48.4 ± 3.5 days (*n* = 9), and the mice treated with capecitabine lived 41.5 ± 8.5 days (*n* = 13). Importantly, the mice treated with diet TC5 did not lose weight (Figure 6b). The results shown in Figure 6b indicated that diets TC6, TC7, and TC8 were worse than diet TC5; the mice fed diet TC6 lived 35.5 ± 2.0 days (*n* = 3), the mice fed diet TC7 lived 33.0 ± 2.5 days (*n* = 3), and the mice fed diet TC8 lived 37.3 ± 7.7 days (*n* = 3). These data indicated that controlling the Gln and Leu levels is crucial for anticancer activity and that the addition of Cys reduced the activity of the diet. However, the inactivity of diets TC6 and TC7 (which contained very low levels of Cys) indicated that Cys restriction was not sufficient to achieve in vivo anticancer activity in colon cancer.

## 4. Discussion

Cancer metabolism has been extensively studied in recent years in an effort to develop better anticancer treatments [7,10,11]. Longstanding and recent evidence indicates that the altered AA metabolism of cancer cells can be therapeutically targeted. For example, L-asparaginase has been used for decades to treat patients with some types of leukemia; this enzyme depletes Asn from the blood and selectively kills leukemia cells that cannot synthesize Asn by themselves [37]. Other AA-depleting enzymes are being investigated, including ADI-PEG20 [38,39], cyst(e)inase [40,41,42], and METase [43,44,45,46], which deplete Arg, Cys, and Met, respectively. Small-molecule drugs that target the metabolism of AAs, such as glutaminase inhibitors [47], Ser-Gly metabolism inhibitors [48,49,50], and Cys transport inhibitors [51,52] are also being developed. Dietary interventions are also growing as a new way to treat cancer [10]. Dietary restrictions of Ser-Gly [21,22,23], Cys [15,53,54], Arg [27,55], Asn [56,57,58], Gln [59], Pro [60], Met [17,18,61,62,63,64], and Leu [65,66] have shown anticancer effects in different types of cancer. In colon cancer, the restriction of Cys and Met [15,16,17,18,19], Ser and Gly [21,22,23,24,25,26], and Arg [27,28,29] induces anticancer effects.

In this work, we took a different approach to target cancer metabolism. Instead of restricting the levels of a particular AA, we sought to create massive changes in AA levels and ratios to generate a challenging metabolic environment for cancer cells. To accomplish this, we eliminated or reduced the levels of many AAs simultaneously (diet TC1 lacked 10 AAs), while increasing the levels of other AAs (e.g., Gln and Leu). As discussed previously [67], cancer cells may be selectively killed by changing the environment under which they have evolved. Briefly, the evolution and survival of cancer cells depend not only on the acquisition of beneficial DNA changes for cancer cells but also on favorable environments for these DNA changes [68]. This means that the DNA changes that provide a survival benefit in a specific environment may be lethal in a different environment. Nutrient availability is a key environmental factor for survival, and all cancer cells have evolved under environments in which the levels and ratios of most nutrients, including AAs, have remained relatively constant; normal diets provide the 20 proteinogenic AAs at relatively constant levels and ratios. By changing the levels and ratios of these AAs, we can create a challenging metabolic environment for cancer cells. For example, although many cancer cells may have mutations in pathways that are involved in the synthesis of specific NEAAs, these mutations will not be lethal to cancer cells if we eat a normal diet that provides adequate levels of all NEAAs. However, if we temporarily change this normal metabolic environment by using an artificial diet lacking NEAAs, we will generate a new metabolic environment that may be lethal to cancer cells. Normal cells would survive because their functional genome would allow them to synthesize the restricted AAs or to enter cell cycle arrest [67].

We used a patient-oriented approach to evaluate the therapeutic potential of this anticancer strategy. Because cancer patients need treatments that are more effective than the existing therapies, this approach is based on establishing suitable in vitro and in vivo conditions to detect whether an experimental treatment is better than the standard treatment used in cancer patients. Selectivity (in vitro) and survival (in vivo) are the parameters used in this patient-oriented approach to compare the efficacy of the experimental treatment with that of the standard anticancer treatment [6].

We began our investigation by preparing an artificial medium that lacked 10 AAs (M1 medium). This medium lacked 10 NEAAs to force cancer cells to biosynthesize them. The medium contained the 9 EAAs and Gln. We included Gln because this AA provides amino groups for the biosynthesis of the other NEAAs. In vitro experiments revealed that this artificial medium was more selective than the standard anticancer drug 5-FU when tested in human colon cancer cells (HT-29) versus human non-malignant cells (HaCaT) (Figure 1 and Figure 2). 5-FU actually lacked selectivity toward cancer cells, probably because the normal cells used in our screening have high proliferative rates and chemotherapy drugs also target normal cells with high proliferative rates. The anticancer drug 5-FU is widely used in patients with metastatic colon cancer and is the active metabolite of the first-line drug capecitabine. Although these in vitro results provide useful information, they should be interpreted cautiously. For example, even though dietary AA restriction can lead to significant in vivo reductions in the levels of the restricted AAs [17,22], the drastic reductions achieved in vitro are difficult to reach in vivo. Furthermore, the metabolic environment of cancer cells grown in vitro is very different from their in vivo metabolic environment [10,69,70]. Therefore, we continued our investigation using in vivo colon cancer models.

We initially prepared a diet lacking the same 10 NEAAs as medium M1, namely, diet TC1. After observing that this diet induced anticancer activity in our initial in vivo screening, we also prepared three TC1-related diets: TC2, TC3, and TC4. Table 2 shows that these diets lacked 9–10 NEAAs and contained high levels of Leu and Gln (mainly to reduce proteolysis and provide sufficient amino groups for AA biosynthesis [67]). These diets contained low lipid levels (1%) because our unpublished data on renal cell carcinoma indicated that lipid restriction maximizes the efficacy of other artificial diets. We used salmon oil as a source of lipids to ensure an adequate supply of essential fatty acids. After carrying out several experiments, we observed that diet TC1 improved mice survival in the intraperitoneal colon cancer model; the mice treated with this diet lived longer than the mice treated with several cycles of capecitabine (Figure 3a). Diet TC1 also improved mice survival in the tail vein colon cancer model, although it did not improve the survival rates achieved with capecitabine (Figure 4a). Diets TC2, TC3, and TC4, which were supplemented with a small amount of Cys (0.2%), were completely inactive (Figure 3c).

Although diet TC1 induced anticancer activity in both colon cancer models and was well tolerated, the mice fed this diet progressively lost weight in the tail vein colon cancer model (Figure 4b). In addition, the poor organoleptic properties of diets based on free AAs may limit their clinical use. To overcome these limitations, we sought to optimize our artificial diets by incorporating small amounts of the protein casein, which contains very low levels of Cys. After observing that 6% casein was the minimum amount of casein required to avoid weight loss in the mice (results not shown), we prepared several casein-based diets: TC5, TC6, TC7, and TC8. In all these diets, all AAs (essential and non-essential) were restricted (not eliminated), except Gln and Leu, which were supplemented in diets TC5, TC7, and TC8. Diet TC5, which contained 6% casein, 5% Gln, and 2.5% Leu, showed marked anticancer activity in both models of colon cancer. The mice fed diet TC5 lived longer than the mice receiving several cycles of the maximum tolerated doses of the first-line drug capecitabine (Figure 5a and Figure 6a). This artificial diet was very well tolerated by the mice in both cancer models, and their body weights remained relatively constant (Figure 5b and Figure 6b). No long-term toxicity was observed in the two mice that survived treatment and were sacrificed on days 342 and 365. A TC5-based diet (also containing 6% casein, 5% Gln, 2.5% Leu, and 1% salmon oil) is currently being evaluated as a monotherapy in a pilot clinical study in patients with metastatic cancers, including colon cancer. Blood tests in these patients will shed light on the metabolic changes elicited by this diet.

Mechanistically, our experiments revealed that the addition of 0.2% Cys to diets TC1 and TC5 blocked or markedly reduced their anticancer activity (Figure 3c and Figure 6c), therefore indicating that Cys restriction was crucial for the anticancer activity of the diets. However, Cys restriction is not sufficient to achieve in vivo anticancer activity in colon cancer because diets TC6 and TC7 (which also contained very low levels of Cys) were inactive. By comparing the in vivo anticancer activity of diets TC5 and TC6 (Figure 5a) and diets TC5, TC6, and TC7 (Figure 6c), we also concluded that controlling the levels of Gln and Leu was important to achieve in vivo anticancer activity with our artificial diets in colon cancer.

The precise mechanism by which diets TC1 and TC5 induced anticancer activity in the mice with metastatic colon cancer is unclear. Our results showed that Cys restriction was necessary to obtain in vivo anticancer activity in colon cancer. Previous studies showed that Cys restriction induces anticancer activity [15,20,40,41,53,71,72], and evidence indicates that Cys-restricted diets reduce Cys availability in vivo [15]. Cys restriction can lead to the accumulation of reactive oxygen species (ROS) in cancer cells by reducing the levels of the tripeptide glutathione (Glu-Cys-Gly) [40,54]; glutathione plays a key role in protecting cells against the lethal effects of reactive oxygen species (ROS) such as hydrogen peroxide and lipid peroxides [15,41]. In addition, since cancer cells have increased basal levels of ROS [73], cancer cells may be more vulnerable than normal cells to therapies that increase ROS levels. These data would contribute to explaining why Cys-restricted diets TC1 and TC5 induced in vivo anticancer activity without causing toxicity in the animals. However, our results showed that cysteine restriction was not sufficient for activity, indicating that other mechanisms were involved.

The present work showed that controlling Leu and Gln levels was important for achieving anticancer activity in vivo. Leu is an essential intracellular sensor of AAs under starvation conditions [74]. Leu activates mTORC1 signaling [74] and inhibits autophagy and proteasome-mediated proteolysis [75,76]. Supplementing Leu may therefore prevent intracellular and extracellular proteolysis [74,77,78]. If muscle and liver proteolysis is not prevented, the lysis of proteins in these organs would supply the AAs that we restrict with our artificial diets. Inhibition of proteolysis is also important for avoiding weight loss and cachexia. On the other hand, Gln is needed for the cellular transport of Leu and other EAAs via LAT1 [79]. Gln also provides amino groups for the biosynthesis of NEAAs (in cells with functional genomes in which these biosynthetic pathways can be activated). These data may contribute to explaining why our active diets require relatively high levels of Leu and Gln.

Since diet TC1 lacked 10 NEAAs and diet TC5 had low levels of all essential and non-essential AAs (except Gln and Leu), we also hypothesize that our diets may inhibit protein synthesis in cells that are unable to obtain sufficient levels of these AAs via biosynthesis or via acquisition from the extracellular environment [80,81]. Several restricted AAs are known to participate in other cellular functions. For example, Met is crucial for metabolism and epigenetics, and its restriction is known to affect cancer cells [82]. Ser and Gly participate in one-carbon metabolism and in nucleotide biosynthesis [22,83], which is essential for cancer cell proliferation. Although the precise mechanism by which diets TC1 and TC5 induce marked anticancer activity in the mice with metastatic colon cancer is unclear, these diets probably create unfavorable metabolic environments for the proliferation and survival of colon cancer cells.

## 5. Conclusions

In this work, we evaluated the therapeutic potential of selective AA restriction in colon cancer. After observing anticancer activity in vitro, we prepared several artificial diets and observed that a diet lacking 10 AAs (diet TC1) and a diet whose only source of AAs was 6% casein, 5% Gln, and 2.5% Leu (diet TC5) improved mice survival in two challenging models of metastatic colon cancer. The mice fed diet TC5 lived longer than the mice treated with several cycles of maximum tolerated doses of capecitabine, which is a drug used in monotherapy as a first-line treatment for patients with metastatic colon cancer. Treatment with diet TC5 was very well tolerated. Mechanistically, Cys supplementation blocked the activity of diets TC1 and TC5, but Cys restriction was not sufficient for activity. Controlling the levels of Gln and Leu was also important for activity. Our results suggest that artificial diets based on the selective restriction of AAs have therapeutic potential in colon cancer.

## 6. Patents

J.J. Jiménez-Alonso, E. Guillén-Mancina, J.M. Calderón-Montaño, V. Jiménez-González, E. Burgos-Morón, and M. López-Lázaro are inventors of a patent related to this research that is licensed to AMINOVITA, S.L., and the University of Seville.

## Figures and Tables

**Table 1:** Composition of M0 (complete medium) and M1 (selective AA restricted medium) in mg/L.

Amino Acid	M0	M1
L-phenylalanine	192	192
L-histidine	80	80
L-lysine	240	240
L-threonine	160	160
L-isoleucine	96	96
L-valine	235	235
L-leucine	528	528
L-tryptophan	16	16
L-methionine	48	48
L-glutamine	1000	1000
L-arginine	100	
Glycine	200	
L-alanine	20	
L-aspartic acid	20	
L-serine	40	
L-tyrosine	100	
L-cysteine dihydrochloride	60	
L-asparagine-1-hydrate	50	
L-glutamic acid	20	
L-proline	20	

**Table 2:** Compositions of the 8 artificial diets used in this work (g/100 g dry diet).

DIET	TC1	TC2	TC3	TC4	TC5	TC6	TC7	TC8
Casein					6	6	6	6
Glutamine (Gln)	2	2	2	2	5		5	5
Leucine (Leu)	3	3	0.6	3	2.5		5	2.5
Methionine (Met)	0.18	0.18	0.18	0.15				
Phenylalanine (Phe)	0.6	0.6	0.6	0.6				
Histidine (His)	0.2	0.2	0.2	0.2				
Lysine (Lys)	0.5	0.5	0.5	0.5				
Threonine (Thr)	0.25	0.25	0.25	0.25				
Isoleucine (Iso)	0.3	0.3	0.3	0.3				
Valine (Val)	0.35	0.35	0.35	0.35				
Tryptophan (Trp)	0.07	0.07	0.07	0.07				
Cystine (CySS)		0.2	0.2	0.2				0.2
Arginine (Arg)								
Glycine (Gly)								
Serine (Ser)								
Tyrosine (Tyr)								
Alanine (Ala)								
Aspartate (Asp)								
Proline (Pro)								
Asparagine (Asn)								
Glutamate (Glu)								
Salmon oil	1	1	1	1	1	1	1	1
Choline	0.25	0.25	0.25	0.25	0.25	0.25	0.25	0.25
Tert-butylhydroquinone	0.0008	0.0008	0.0008	0.0008	0.0008	0.0008	0.0008	0.0008
Vitamin Mix	1	1	1	1	1	1	1	1
Mineral Mix	3.5	3.5	3.5	3.5	3.5	3.5	3.5	3.5
Sucrose	15	15	15	15	15	15	15	15
Cellulose	5	5	5	5	5	5	5	5
Corn starch	66.80	66.60	69.00	66.63	60.75	68.25	58.25	60.75
Total	100	100	100	100	100	100	100	100

## Data Availability

Not applicable.

## Supplementary Materials

The following supporting information can be downloaded at: https://www.mdpi.com/article/10.3390/nu14163378/s1. Figure S1: Liver and spleen weights of the mice fed diets T1–T4; Figure S2: Liver and spleen weights of the mice fed diets T5–T8; Figure S3: Representative photographs at the time of sacrifice of the mice with colon cancer in the peritoneal dissemination model; Figure S4: Lung photographs at the time of sacrifice of representative mice with colon cancer in the lung metastasis model; Figure S5: Photographs of a mouse fed diet TC1 that survived treatment; Figure S6: Photographs of a mouse treated with capecitabine that survived treatment; Figure S7: Photographs of a representative mouse fed diet TC5 that survived treatment.
