# Supplementary material for: Artificial Diets Based on Selective Amino Acid Restriction versus Capecitabine in Mice with Metastatic Colon Cancer
Source: Nutrients. 2022 Aug 17;14(16):3378. doi: 10.3390/nu14163378 (PMC9412877; doi:10.3390/nu14163378)
Supplement: Supplementary file 1 [file nutrients-14-03378-s001.zip › nutrients-1875187-supplementary.pdf]

nutrients

MDPI

Article

Artificial Diets Based on Selective Amino Acid Restriction versus Capecitabine in Mice with Metastatic Colon Cancer

Julio José Jiménez-Alonso[1#], Emilio Guillén-Mancina[1#], José Manuel Calderón-Montaño[1#], Víctor Jiménez-González[1], Patricia Díaz-Ortega[1], Estefanía Burgos-Morón[1], Miguel López-Lázaro[1*]

[1] Department of Pharmacology, Faculty of Pharmacy, University of Seville, Sevilla, Spain
* Correspondence: Miguel López-Lázaro. Department of Pharmacology, Faculty of Pharmacy, C/Profesor Garcia Gonzalez 2, Sevilla, 41012, Spain. E-mail: mlopezlazaro@us.es Tel: +34-954-55-63-48, https://orcid.org/0000-0003-2794-1647
\# J.J. Jiménez-Alonso, E. Guillén-Mancina and J.M. Calderón-Montaño contributed as co-first authors of this article

Citation: Citation: Jiménez-Alonso J.J.; Guillén-Mancina E.; Calderón-Montaño J.M.; Jiménez-González V.; Díaz-Ortega P.; Burgos-Morón E.; López-Lázaro M. Artificial Diets Based on Selective Amino Acid Restriction versus Capecitabine in Mice with Colon Cancer. *Nutrients* **2022**, *14*, 3378. https://doi.org/10.3390/nu14163378

Academic Editors: Evasio Pasini, Francesco S. Dioguardi and Giovanni Corsetti

Received: 3 August 2022
Accepted: 14 August 2022
Published: 22 August 2022

Publisher's Note: MDPI stays neutral with regard to jurisdictional claims in published maps and institutional affiliations.

Copyright: © 2022 by the authors. Licensee MDPI, Basel, Switzerland. This article is an open access article distributed under the terms and conditions of the Creative Commons Attribution (CC BY) license (https://creativecommons.org/licenses/by/4.0/).

1. Supplementary materials

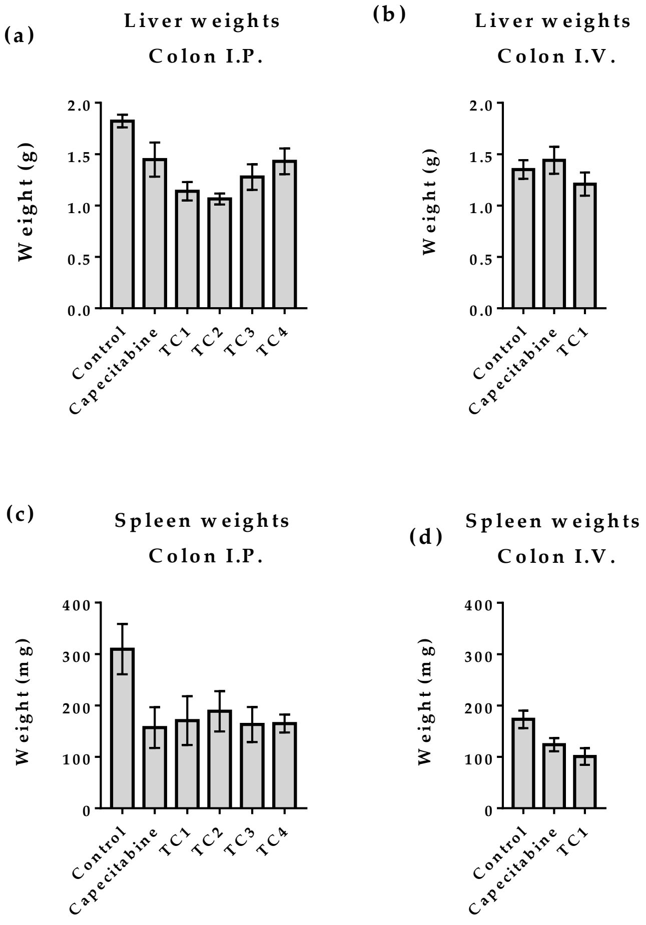

Figure S1. Liver and spleen weights of mice fed diets T1-T4. In the colon intraperitoneal (I.P.) model, CT26.WT murine colon cancer cells were inoculated in the peritoneum of immunocompetent

BALB/c mice. In the colon intravenous (I.V) model, CT26.WT murine colon cancer cells were inoculated in the tail vein of immunocompetent BALB/c mice. Organs were excised and weighted at the time of sacrifice (see text for details).

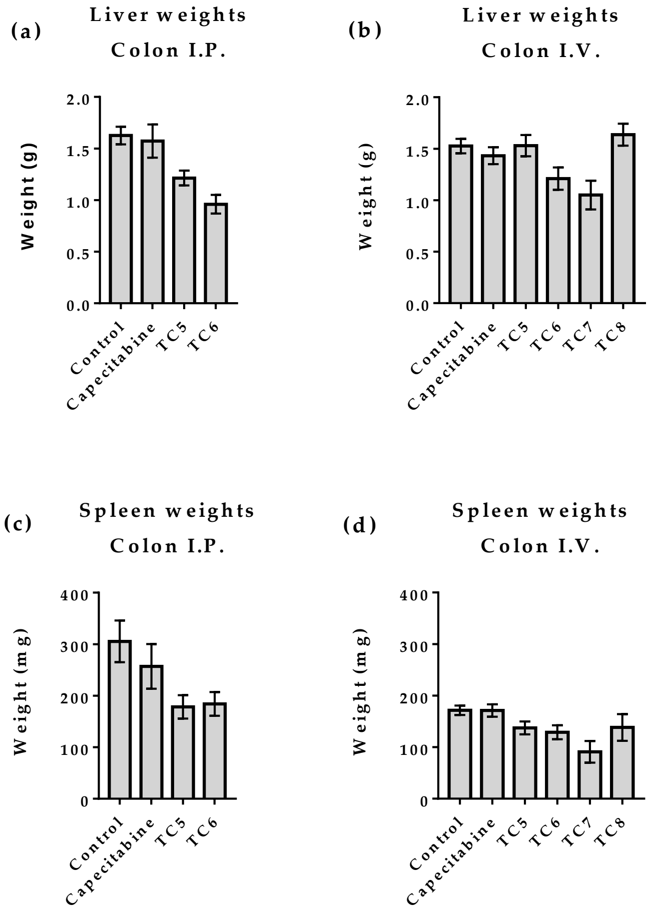

Figure S2. Liver and spleen weights of mice fed diets T5-T8. In the colon intraperitoneal (I.P.) model, CT26.WT murine colon cancer cells were inoculated in the peritoneum of immunocompetent BALB/c mice. In the colon intravenous (I.V) model, CT26.WT murine colon cancer cells were inoculated in the tail vein of immunocompetent BALB/c mice. Organs were excised and weighted at the time of sacrifice (see text for details).

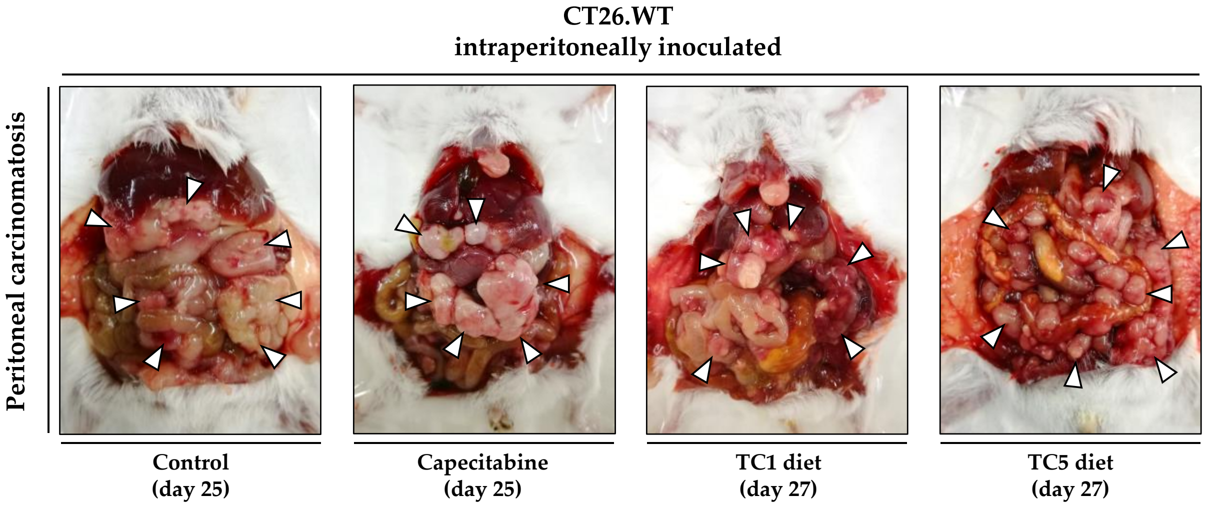

Figure S3. Representative photographs at the time of sacrifice of mice with colon cancer in the peritoneal dissemination model. In this model, 100 000 CT26.WT murine colon cancer cells are inoculated in the peritoneum of immunocompetent BALB/c mice. Each mouse was sacrificed at different time points, when symptoms of advanced disease were patent. The day of sacrifice is shown in brackets.

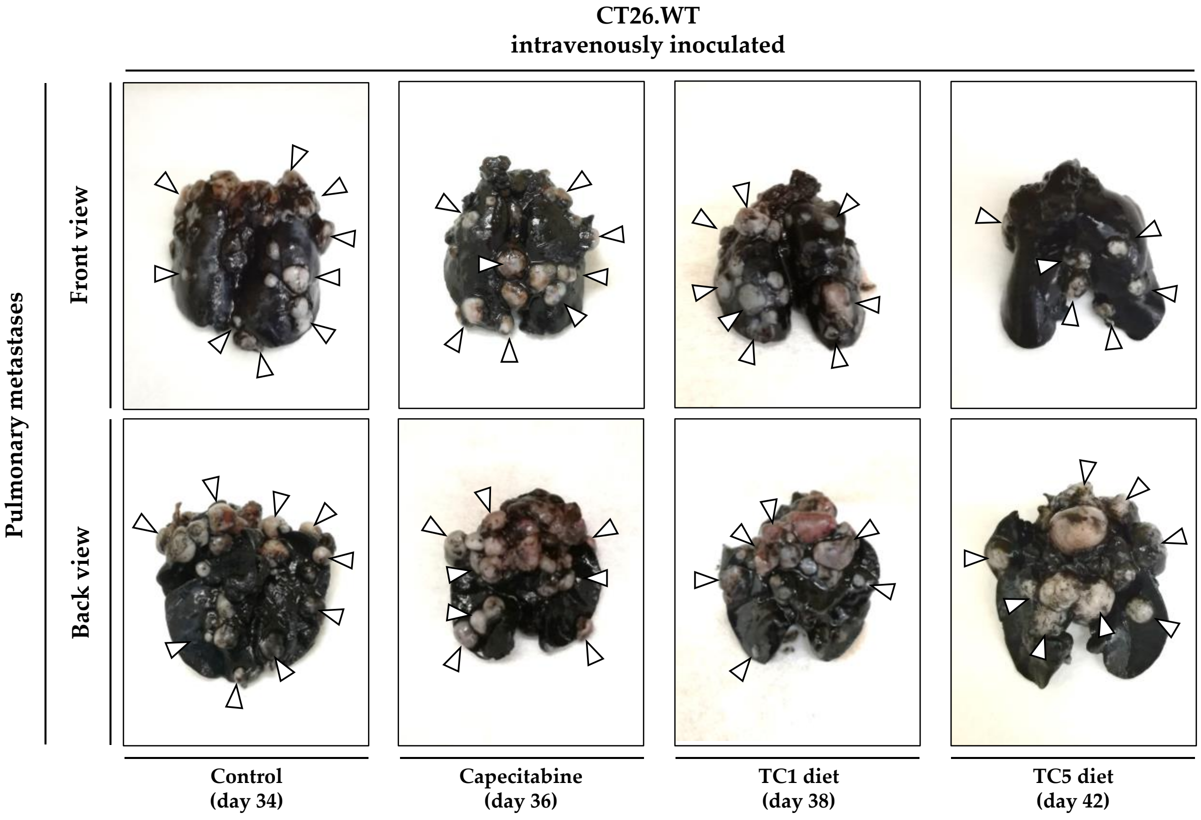

Figure S4. Lung photographs at the time of sacrifice of representative mice with colon cancer in the lung metastasis model. In this model, 100 000 CT26.WT murine colon cancer cells are inoculated in the tail vein of immunocompetent BALB/c mice. After sacrifice, lungs were excised and stained with India ink (tumors show a white appearance and normal lung parenchyma appears black). Each mouse was sacrificed at different time points when symptoms of advanced disease were patent. The day of sacrifice is shown in brackets.

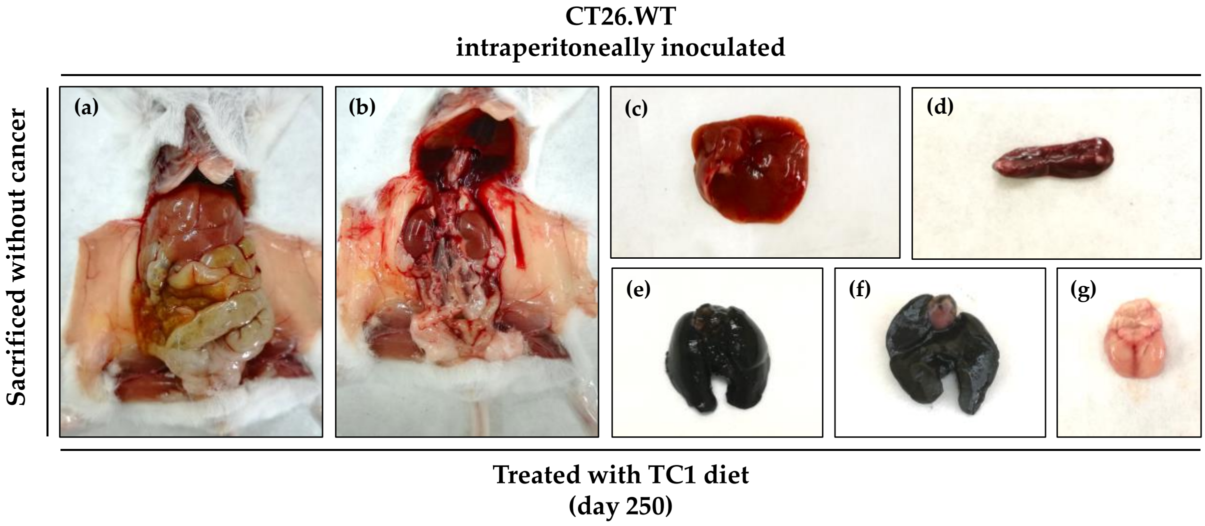

Figure S5. Photographs of mouse fed diet TC1 that survived treatment. This mouse was sacrificed 250 days after the intraperitoneal inoculation with CT26.WT colon cancer cells. No tumors were found. (**a**) Peritoneal cavity. (**b**) Retroperitoneal space, kidneys, and diaphragm. (**c**) Liver. (**d**) Spleen. (**e**) Front view of lungs dyed with India ink. (**f**) Back view of lungs dyed with India ink (and heart). (**g**) Brain.

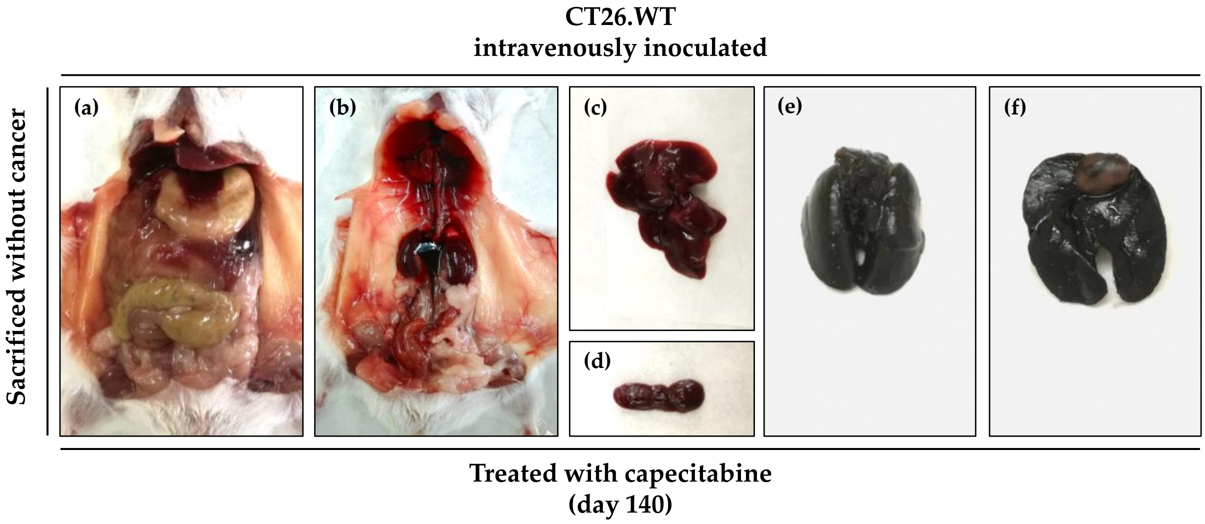

Figure S6. Photographs of mouse treated with capecitabine that survived treatment. This mouse was sacrificed 140 days after intravenous inoculation of CT26.WT colon cancer cells. No tumors were found. (**a**) Peritoneal cavity. (**b**) Retroperitoneal space, kidneys, and diaphragm. (**c**) Liver. (**d**) Spleen. (**e**) Front view of lungs dyed with India ink. (**f**) Back view of lungs dyed with India ink (and heart).

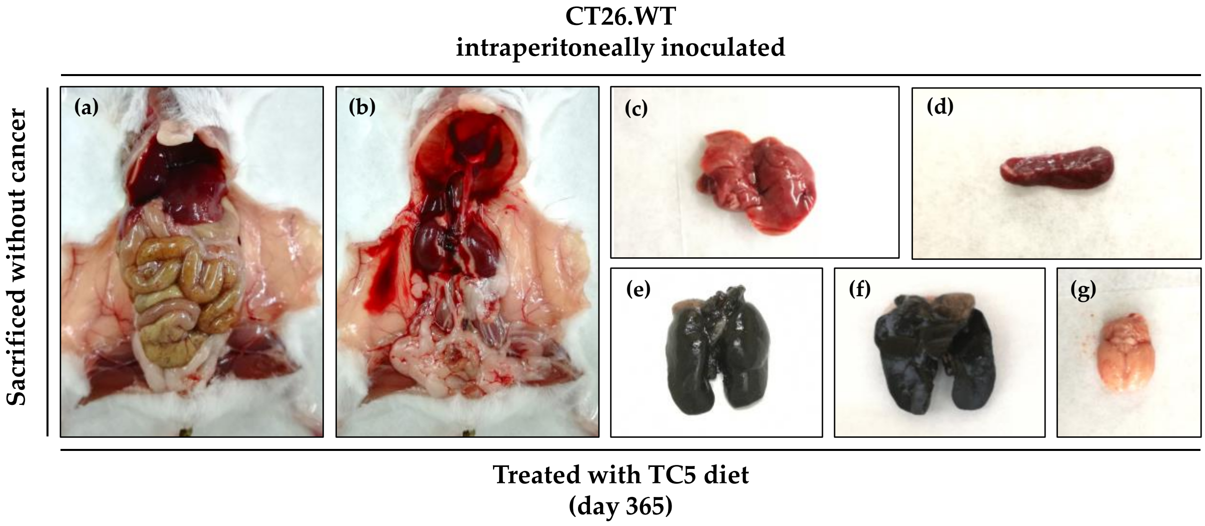

Figure S7. Photographs of a representative mouse fed diet TC5 that survived treatment. This mouse was sacrificed 365 days after the intraperitoneal inoculation of CT26.WT colon cancer cells. No tumors were found. (**a**) Peritoneal cavity. (**b**) Retroperitoneal space, kidneys, and diaphragm. (**c**) Liver. (**d**) Spleen. (**e**) Front view of lungs dyed with India ink. (**f**) Back view of lungs dyed with India ink (and heart). (**g**) Brain.
